# The Importance of the Transcription Factor Foxp3 in the Development of Primary Immunodeficiencies

**DOI:** 10.3390/jcm11040947

**Published:** 2022-02-11

**Authors:** Paulina Mertowska, Sebastian Mertowski, Martyna Podgajna, Ewelina Grywalska

**Affiliations:** Department of Experimental Immunology, Medical University of Lublin, Chodźki 4a St., 20-093 Lublin, Poland; paulinamertowska@umlub.pl (P.M.); marpodgajna@gmail.com (M.P.); ewelina.grywalska@umlub.pl (E.G.)

**Keywords:** Foxp3, IPEX, CVID, primary immunodeficiencies

## Abstract

Transcription factors are an extremely important group of proteins that are responsible for the process of selective activation or deactivation of other cellular proteins, usually at the last stage of signal transmission in the cell. An important family of transcription factors that regulate the body’s response is the FOX family which plays an important role in regulating the expression of genes involved in cell growth, proliferation, and differentiation. The members of this family include the intracellular protein Foxp3, which regulates the process of differentiation of the T lymphocyte subpopulation, and more precisely, is responsible for the development of regulatory T lymphocytes. This protein influences several cellular processes both directly and indirectly. In the process of cytokine production regulation, the Foxp3 protein interacts with numerous proteins and transcription factors such as NFAT, nuclear factor kappa B, and Runx1/AML1 and is involved in the process of histone acetylation in condensed chromatin. Malfunctioning of transcription factor Foxp3 caused by the mutagenesis process affects the development of disorders of the immune response and autoimmune diseases. This applies to the impairment or inability of the immune system to fight infections due to a disruption of the mechanisms supporting immune homeostasis which in turn leads to the development of a special group of disorders called primary immunodeficiencies (PID). The aim of this review is to provide information on the role of the Foxp3 protein in the human body and its involvement in the development of two types of primary immunodeficiency diseases: IPEX (Immunodysregulation Polyendocrinopathy Enteropathy X-linked syndrome) and CVID (Common Variable Immunodeficiency).

## 1. Introduction

Transcription factors are a group of proteins that exhibit the ability to bind to genetic material (DNA). The site of attachment of transcription factors in the DNA region may be a promoter or an enhancer sequence in a specific site or region that regulates the transcription process. The action of transcription factors can be selectively activated in the cell or deactivated by other cellular proteins which usually takes place at the last stage of signal transmission in the cell [1]. One of the more important families among transcription factors is the FOX family, which plays an important role in regulating the expression of genes involved in cell growth, proliferation, and differentiation. This family includes the Foxp3 protein (Forkhead box protein P3) which is not only a transcription factor but also a key molecule involved in the development of regulatory T cells (Treg) [2,3]. It plays an important role in maintaining the homeostasis of the immune system, enables the complete stability of the Treg lineage, and directly modulates the expansion and function of conventional T-cells [3,4]. The Foxp3 protein can act both as a repressor and activator of the transcription process, while the type of its activity depends on its interaction with other transcription factors present in the cell [4]. Disturbances in the proper functioning of the Foxp3 protein in the human body (usually caused by mutations within the coding sequences) dysregulate the immune homeostasis and leads to the development of diseases called immunodeficiencies [5,6]. This is a special group of disorders in which the patient’s immune system is characterized by a reduced ability or lack of defense against various pathogens. It is manifested by frequent, recurrent infections with microorganisms (bacteria, viruses, fungi) that are resistant to long-term therapy [7]. In the vast majority of cases it is caused by microbes commonly inhabiting the human body which, as a result of weakened immune reactions, cause the so-called opportunistic infections [8]. Apart from the tendency of recurrent infections, immunodeficiency is also accompanied by other health problems such as allergic phenomena [9], autoimmune phenomena [10,11], granulomas [12], tumors [13,14], endocrine disorders [15,16], and various cytopenias (most often thrombocytopenia [17] and neutropenia [18,19]) as well as diseases of the lungs [20] and gastrointestinal tract [21] (e.g., irritable bowel syndrome) [22,23]. Due to the underlying causes of immunodeficiencies, we can divide them into two groups: Primary Immunodeficiencies (PID), which are genetically determined and associated with mutations that are either inherited or appear de novo and Secondary Immunodeficiencies (SID), which are caused by external factors or the presence of comorbid disease [24,25].

The aim of this review is to present the role of the Foxp3 transcription factor in the human body in maintaining immune homeostasis and its significance during the development of immunodeficiency. Due to the Foxp3 protein’s involvement in the regulation of transcription, we will try to explain its role in the development of primary immunodeficiency diseases that are strongly associated with genetic disorders.

## 2. Molecular Characterization of the Foxp3 Protein

**Figure 1:**
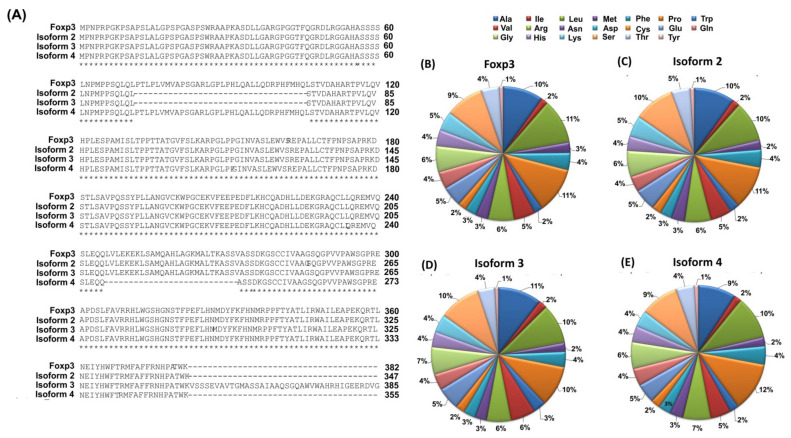
Characterization of the amino acid sequence of the Foxp3 protein and its isoforms (**A**) Comparison of the amino acid sequence of the Foxp3 protein and its isoforms; (**B**–**E**) Amino acid scald of Foxp3 protein and its isoforms [own elaboration]. Marks: (*) means the amino acids are identical in the sequence, while (-) means the lack of amino acids in the sequence.

The human *FOXP3* gene is located on the X chromosome, more specifically, by genomic sequence analysis, it was shown to be on the p arm at position Xp11.23 [26,27]. The gene is composed of 12 exons whose exon-intron boundaries are identical in the gene coding regions of both mice and humans [28]. The degree of nucleotide sequence identity between these organisms is 84.98% [26,27,28]. The *FOXP3* gene is responsible for the encoding of a protein product composed of 431 amino acids with a total molecular weight of 47.27 kDa and an isoelectric puncture of 8.62 (Table 1). The analysis of the protein sequence in the Foxp3 protein showed that its structure contains 47.33% hydrophobic amino acids and 52.67% hydrophilic amino acids (Table 1). According to the literature, the Foxp3 protein can also occur in the human body in the form of three other isoforms: isoforms 2, 3, and 4 [29]. They differ mainly in amino acid length and molecular weight which changes the amino acid composition, isoelectric point, and secondary structure of the protein. Our research team performed a detailed analysis of the similarity of the amino acid sequence of the Foxp3 protein and its three isoforms using the UniProt database. The differences made on this basis are shown in Table 1 and Figure 1.

For isoform 2, 34 amino acids are missing from the Foxp3 protein sequence (located at positions 72–106). The same is the case for isoform 3 where at position 382, a fragment consisting of 61 amino acids (-KVSSSEVAVTGMASSAIAAQSGQAWVWAHRHIGEERDVGCWWWLLASEVDAHLLPVPGLPQ-) is also added [29]. Additionally, between the Foxp3 protein and isoform 4, there is a difference of 26 amino acids located between amino acids 246–272 which determines the highest degree of sequence identity. The degree of identity of the amino acid sequence of the Foxp3 protein and its isoforms is quite diverse and ranges from 75.15% (between isoforms 3 and 4) to 93.73% (between the Foxp3 protein and isoform 4). Detailed information on the degree of amino acid sequence identity between individual proteins is provided in Table 2.

Foxp3 isoforms differ not only in their structure and the degree of similarity of the amino acid sequence but also in their ability to interact with different proteins. The Foxp3 protein is able to interact with the IKZE3 protein (via the LXXLL motif), while as reported in the literature, isoform 2 does not [4,30]. It has been shown that it is able to interact with the ZFP90 protein and create complexes with the TRIM28 protein [31]. However, further research is needed to understand the differential function of Foxp3 isoforms.

**Figure 2:**
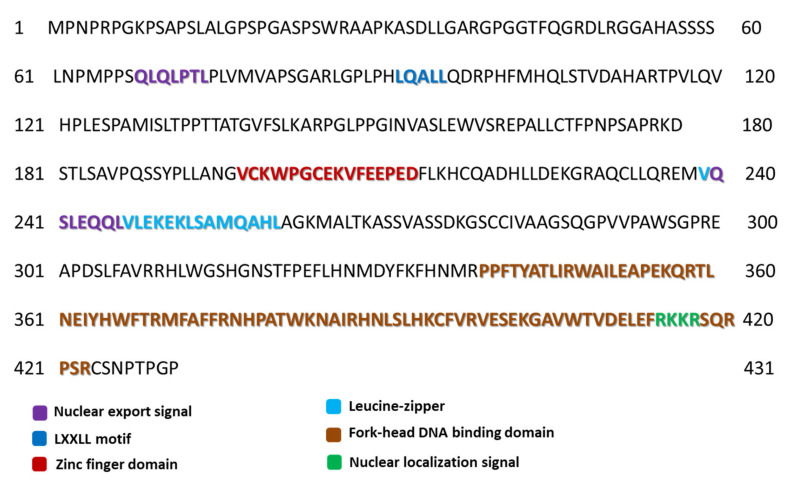
Motifs and domains occurring in the amino acid sequence of the Foxp3 protein (own elaboration based on the Uniprot database [29]).

The Foxp3 protein has several distinctive motifs in its structure. Two of them, located between amino acids 68–76 and amino acids 239–248, are nuclear export signals which are short peptides containing hydrophobic residues targeted for export from the cell nucleus into the cytoplasm through the nuclear pore complex (Figure 2) [32]. Another example is the LXXLL motif, located between amino acids 92–96, which is involved in many protein-protein interactions related to various aspects of transcription regulation (Figure 2) [33,34,35]. These motifs are present in many transcription factors and cofactors, mediating interactions that may activate or suppress transcription [36,37]. Several recently reported 3D structures of protein-LXXLL motif complexes have been associated with leukemia, further highlighting the diversity and regulatory importance of this seemingly simple motif [36,38,39].

Another motif located between amino acids 414–417 is the nuclear localization signal which is an amino acid sequence that “tags” a protein for import into the cell nucleus via nuclear transport (Figure 2). Typically, this signal consists of one or more short sequences of positively charged lysines or arginines exposed on the protein surface [40]. Two extremely important domains within the sequence of this protein should also be noted. The first is a zinc finger domain of 26 amino acids located in the region between amino acids 197 and 222 (Figure 2). It is a structure found in DNA binding proteins and is directly involved in the binding of a nucleic acid molecule by a protein [41]. The presence of the zinc ion (Zn^2+^) in the domain is crucial for the stability of the entire structure as its absence may result in structural and functional changes. The zinc atom in this structure is coordinated by residues C198, C203, H216, H221, and partially by D220 [42,43]. Near the zinc finger domain, there is another leucine zipper motif (239–260 amino acids) which is very important due to the functions performed by the Foxp3 protein [44,45]. This motif mediates intermolecular interactions, indicating the possible interaction in the dimerization process which is the essential function of the transcriptional regulator [45]. As indicated by research data, this motif is involved not only in homo-association but also in hetero-association with the Foxp1 protein [44,45]. Additionally, it has been shown that the presence of mutations within the leucine zipper motif can significantly reduce the binding affinity of the Foxp3 protein to the promoter regions in vitro [42]. The discoveries made by Mackey-Cushman et al., (2011) showed that the leucine zipper motif also mediates interactions between the Foxp3 protein and histones (the H1.5 histone, precisely) which suppress interleukin 2 (IL-2) transcription in T cells [46]. It should also be mentioned that the zinc finger motif is not directly involved in the dimerization process and the very mechanism of this process is not fully understood [42]. The second extremely important structure is the domain called “fork-head”, which is often described as the transcription factor, whose purpose is to bind DNA. In the case of the Foxp3 protein, this domain is 87 amino acids long and is located between amino acids 337–423 in the sequence (Figure 2) [47,48]. It is involved in the protein dimerization process and its interaction with nuclear factor NFATC2 is responsible for activating T cells [49]. The NFATC2 protein is present in the cytosol and translocates only to the nucleus upon stimulation of the T-cell receptor (TCR) where it becomes a member of the nuclear factors of the activated T-cell transcription complex. This complex plays a key role in inducing gene transcription during the immune response [49,50].

## 3. The Occurrence, Interactions, and Importance of the Foxp3 Protein in the Human Body

### 3.1. The Occurrence of Foxp3 Protein on Cells of the Immune System and Its Functions in the Human Body

Treg cells belong to the subpopulation of lymphocytes responsible for suppressing an overly increased or autoreactive immune response, which may be specific or non-specific for a given antigen, without causing general immunodeficiency [51,52]. It is an extremely heterogeneous population with several subpopulations of cells with different levels of Foxp3 protein expression (Table 3) [53]. Treg cells expressing Foxp3+ can also be divided into two smaller subpopulations according to the origin of the cells. The first one is natural Treg lymphocytes (nTreg), which are formed in the thymus as a separate developmental line, while the second is induced (adoptive) Treg lymphocytes (iTreg or aTreg) [54,55]. The latter are Treg cells arising in peripheral tissues which initially do not express the Foxp3 factor but acquire it along with suppressive properties upon stimulation with an appropriate antigen. Natural nTreg lymphocytes express CTLA-4 (cytotoxic T cell antigen 4) [56], GITR (glucocorticoid-induced TNFR-related protein) [57], CCR4 (CC chemokine receptor type 4) [58,59], and CD62L (L-selectin) [60]. The formation of nTreg and iTreg lymphocytes requires the presence of interleukin 2 (IL-2) and transforming growth factor beta 1 (TGF-β) [61]. The similarities in the role of these cytokines in the maintenance and survival of both Treg lymphocyte populations are crucial. nTregs develop in response to contact with intrinsic antigens in the thymus and require high affinity between these antigens and MHC complexes [62,63]. This is probably because they arise from continuously proliferating precursor cells. They also require costimulation with CD28 (cluster of differentiation 28) which also plays an important role in the process of inducing the expression of the CTLA-4 molecule in T lymphocytes which can inhibit the CD28 signal and thus, is responsible for the negative feedback mechanism [64,65]. On the other hand, iTreg, through interactions with environmental antigens presented by dendritic cells in peripheral lymphoid organs and their conversion to iTreg CD25+Foxp3+, requires weaker, incomplete TCR stimulation [66]. Foxp3 protein is also known to convert naive T cells into Treg cells that are capable of suppression in vivo and in vitro, suggesting that Foxp3 can regulate the expression of suppression-mediating molecules [53]. Elucidation of Foxp3 gene targets may be crucial for understanding the Treg cell suppressive capacity [67,68,69].

Another group of immune system cells in which the Foxp3 protein is expressed is NKT cells [71]. This is a group of innate lymphocytes capable of producing cytokines characteristic for a Th1, Th2, or Th17 response [72,73]. They have also been confirmed to influence adaptive immunity by exacerbating or suppressing a variety of immune disorders such as autoimmunity and allergy. As mentioned before, Treg cells are characterized by strong immunosuppressive properties and expression of the Foxp3 transcription factor and constitute a key element in maintaining immune homeostasis in the human body. Studies from recent years have shown that NKT cells, like Treg cells, contribute to the maintenance of immune tolerance and are also capable of the Foxp3 protein expression. From animal model studies, scientists were able to establish that NKT cells in the lymph nodes of α-galactoceramide-stimulated mice increased the ability to express Foxp3 in response to TGF-β. However, further research is needed to use Foxp3+ NKT cells for therapeutic purposes in the treatment of immune response disorders [74,75].

In addition to T cells, expression of the Foxp3 protein is also observed on B cells. Regulatory B cells, which are characterized by the production of anti-inflammatory cytokines (e.g., IL-10 and IL-35), also contribute to the enhancement of the immune homeostasis in the human body [74]. Recent research has shown that some B cells are also capable of expressing the Foxp3 protein [76]. They are usually observed in patients diagnosed with multiple sclerosis [77] or in patients with systemic lupus erythema where their increased amount correlates with disease progression [78,79]. A study by Slobodin et al. (2010) shows that the percentage of Breg cells in the peripheral blood of SLE patients is higher than that of healthy controls and that Bregs have been shown to be functionally impaired. Additionally, they showed that with the expansion of CD25highIL-10highFoxP3high B regulator cells, increased disease activity occurs [80]. However, the current literature reports do not provide important information regarding the exact role of the Foxp3 protein on B lymphocytes and its role in the regulation of inflammation [70,81]. The subject of Foxp3 protein expression on cells of the immune system is quite debatable and requires a lot of research to fully understand the importance and role of this protein in the human body.

### 3.2. Interactions of Foxp3 Protein with Proteins and Transcription Factors

The Foxp3 protein plays an important role in the regulation of cytokine production through interaction with numerous proteins and transcription factors such as NFAT, nuclear factor kappa B (NF-κB, kappa-light-chain-enhancer of activated B cells) [82], and Runx1/AML1 (Runt-related transcription factor 1/acute myeloid leukemia 1 protein), also known as acute myeloid leukemia 1 protein (AML1) [83] or alpha-core binding factor 2 (CBFA2) subunit [84]. Transcription factors NFAT and Runx1/AML1 are necessary for the production of IL-2 following TCR receptor stimulation. The association of the Foxp3 protein with these factors inhibits the expression of IL-2, IL-4, (which are pro-inflammatory cytokines), and IFN-γ [85]. In vivo, the transcription factor NFAT, which is required to bind to the proximal region of the promoter for IL-2, participates in direct interactions with the fork-head domain of the Foxp3 protein [82]. These interactions are also necessary to stimulate the expression of some Treg lymphocyte antigens, such as CD25 or CTLA-4 [82,86,87]. CD25 is the alpha chain of the interleukin 2 receptor and is transiently expressed on activated T and B lymphocytes and constitutively present on Treg cells. The presence of this protein was also found on dendritic cells, fibroblasts, and endothelial cells [88,89]. In contrast, CTLA-4 is a protein receptor that acts as a checkpoint for immune responses. This protein is constitutively expressed in Treg lymphocytes, but when activated, it is only up-regulated in conventional T lymphocytes; this phenomenon is particularly evident in the case of neoplastic disorders [86,90]. The interaction of the Foxp3 protein with the Runx1/AML1 factor (association between the “fork-head” domain and the leucine zipper) consists of the attachment of this complex to the IL-2 promoter region. It should also be noted that the Foxp3 protein also participates in interactions with other members of the FOX family where it forms complexes capable of inhibiting the expression of selected genes [91]. The formation of either homodimeric or heterodimeric Foxp3 protein complexes occurs when a leucine zipper is used which associates with the IL-2 promoter in vivo. Scientific studies have also shown that the multimerization of the Foxp3 protein is extremely important for the proper functioning of Treg lymphocytes [92,93,94,95].

The N-terminus of the Foxp3 protein also has properties to inhibit the transcriptional activity of the NFAT factor and is necessary for the inhibition of IL-2 production by T cells. Studies have shown that the association of the Foxp3 protein with the IL-2 and IFN-γ promoters correlates with the process of hypoacetylation (low level of acetylation) of histones in condensed chromatin [4,96]. The binding of the Foxp3 protein to the CD25 and CTLA-4 promoter sequences, with the simultaneous hypoacetylation of condensed chromatin histones, suggests a direct mechanism of transcription activation [97,98,99].

In Treg cells, the Foxp3 protein is present as a part of a large complex that also includes histone acetyltransferases (HAT) and histone deacetylases (HDAC) [100,101]. The process of acetylation and deacetylation is one of the post-translational modifications of histones, involving lysine residues located at the N-terminus (protruding from the nucleosome core), which is an epigenetic mechanism for the control of gene expression. The enzymes catalyzing the reversible acetylation of histones are HAT and HDAC [102]. Research conducted in recent years has allowed us to establish that the former simultaneously act as coactivators of transcription, while the latter is its corepressors. Thus, it is possible to prove the relationship between the covalent modification of chromosomal proteins (acetylation of core histones) and gene expression. HAT and HDAC enzymes are responsible for the acetylation process of the Foxp3 protein and thus determine the functions of this transcription factor. Studies have shown that Foxp3 acetylation is related to its function in Treg cells [103,104,105]. Detailed analyzes have shown that the acetylation process of this protein is influenced by molecules such as KAT5 (TIP60), p300, HDAC6, HDAC7, HDAC9, HDAC10, and SIRT1 (Table 4).

Moreover, it has been shown that the stability of the Foxp3 protein is significantly higher in the presence of the enzyme p300 than in the presence of KAT5. After combining with the p300 protein, Foxp3 shows a higher affinity for DNA chromatin, while after interaction with KAT5, its affinity for the IL-2 promoter has significantly increased [106]. The importance and role of HDAC7 and HDAC9 in the acetylation of Foxp3 protein are poorly understood. The link is due to the fact that class II HDAC has no intrinsic functional catalytic activity and possibly, as the scientists suggest, works by recruiting class I HDAC into the complex. The mechanism of action of HDAC6, belonging to class II HDAC proteins, is based on the regulation of the acetylation process of many cellular proteins, among which α-tubulin and HSP90 deserve special attention [107,108]. Studies conducted by Zoeten et al., showed that the pharmacological inhibition or the use of a genetic knockout of the HDAC6 gene increased the level of acetylation of both Foxp3 and Hsp90 [107] which resulted in an increase in the immunosuppressive activity of Treg cells. According to the researchers, this process may be used in the future to suppress autoimmunity caused by Treg and be used in the prevention of transplant rejection [107,108]. The combination of KAT5 and HDAC7 is an essential mechanism in the inhibition of IL-2 transcription by the Foxp3 protein [100,109]. NAD-dependent sirtuin-1 deacetylase is considered a negative regulator of the acetylation process of the Foxp3 protein [55,110].

### 3.3. The Importance of Post-Transcriptional and Post-Translational Modifications in the Function of the Foxp3 Protein

**Figure 3:**
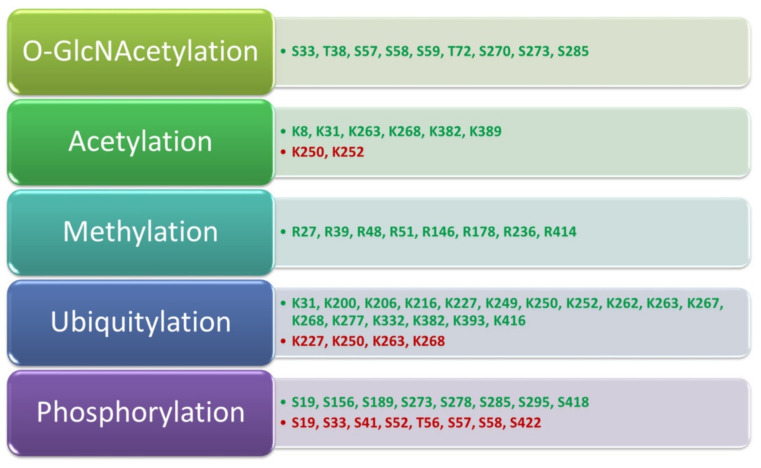
Contribution of PTM to the functioning of the Foxp3 protein and the suppressor functions of Treg lymphocytes. Modifications that positively affect the suppressor functions of Treg lymphocytes are marked in green while those that have a negative effect are in red; based on [105].

The transcription factor Foxp3 is a major regulator of Treg cells’ growth and suppression activity. As indicated in the literature, it is also subject to complex regulation by the participation of many posttranscriptional modifications as well as posttranslational modifications (PTM) which also indirectly affect Treg suppressor activity. The first modifications of the Foxp3 protein concern epigenetic changes related to the regulation of DNA methylation [111], histone modification [99], or nucleosome positioning [112]. In the literature, we can find reports on the regulation of the Foxp3 protein by conserved non-coding sequences 2 (CNS 2) [113,114] or the share of ubiquitin-specific peptidase 22 (USP22) [115,116]. Many factors also play an important role in regulating the conversion of Foxp3 precursor messenger RNA transcripts at the level of post-transcription modification. This concerns the participation of microRNAs (miRNAs), including miR-24, miR-31, and miR-210, the activity of which leads to the degradation of Foxp3 mRNA which consequently prevents the translation of Foxp3 [117,118]. PTM, which includes phosphorylation, O-GlcNAcylation, acetylation, ubiquitylation, or methylation, also significantly influences the activity of the Foxp3 protein [105,119]. It should be noted that most of the PTMs refer to enzymatic processes that are designed to change the protein after its synthesis. Moreover, all the induced modifications affect the characteristics of the protein, including its location and interactions. This is also the case with the Foxp3 protein, in which the occurring PTMs affect the very structure of the protein (its stabilization or degradation) and its interactions with other proteins as well as reducing or increasing the activity of suppressive Treg lymphocytes [105]. Detailed information on the positive and negative contribution of individual PTMs to the suppressor functions of Treg cells is presented in Figure 3.

**Table 4:** Effect of enzymes on the acetylation process of the Foxp3 transcription factor.

Name of the Enzyme	Abbreviation	Functions	Reference
Histone acetyltransferase KAT5	TIP60	Performs histone acetylation in the nucleosome which changes the binding to DNA. Acetylation neutralizes the positive charge on the histones, reducing the binding affinity of negatively charged DNA. This in turn reduces the steric hindrance of DNA and increases the interaction of transcription factors and other proteins. The three key functions of KAT5 are its ability to regulate transcription, DNA repair, and apoptosis.	[30,120]
Histone acetyltransferase	p300	Acts as a histone acetyltransferase that regulates transcription through chromatin remodeling and is important in cell proliferation and differentiation. It mediates the regulation of the cAMP gene, binding specifically to the phosphorylated CREB protein, and also contains a bromodomain which is involved in IL6 signaling.	[106,121]
Histone deacetylase 6	HDAC6	This enzyme is located in the cytoplasm where it is responsible for the regulation of acetylation of α-tubulin, HSP90, or glucocorticoid receptors. Upon activation of Treg cells, this enzyme migrates to the cell nucleus where it participates in the regulation of the acetylation level of the Foxp3 protein. Pharmacological inhibition or the use of a genetic knockout of the HDAC6 gene have been shown to increase the level of acetylation of both Foxp3 and Hsp90 proteins which results in an increase in the immunosuppressive activity of Treg cells.	[107,108,122]
Histone deacetylase 7	HDAC7	HDAC7 has been shown to have low intrinsic deacetylase activity and studies have demonstrated that HDAC7 may have a variety of alternative developmentary, proliferative, and inflammatory functions.	[101,102,123]
Histone deacetylase 9	HDAC9	Represses the activity of MEF2 by recruiting multi-component complexes containing CtBP and HDAC. May play a role in the process of hematopoiesis.	[108,124]
Histone deacetylase 10	HDAC10	From studies performed in a mouse model, HDAC10 deletion did not adversely affect the health of mice that retained normal CD4+ and CD8+ T cell function. However, HDAC10^−/−^ Treg showed enhanced suppressive function both in vitro and in vivo. In addition, HDAC10^−/−^ mice that received a heart transplant with a completely mismatched MHC became more tolerant and showed longer allograft survival.	[101]
NAD-dependent sirtuin-1 deacetylase	SIRT1	SIRT1 deacetylates and thus inactivates the p53 protein. SIRT1 also stimulates autophagy by preventing the acetylation of proteins (via deacetylation) required for autophagy, as demonstrated in cultured cells and embryonic and neonatal tissues. This feature provides a link between sirtuin expression and the cellular response to nutrient constraints due to caloric restriction. SIRT1 inhibits NF-κB regulated gene expression by deacetylating the RelA/p65 subunit of NF-κB in lysine 310. SIRT1 plays a role in activating T17 helper cells that contribute to autoimmune disease.	[125,126]

### 3.4. Importance of T Cell Metabolic Factors and the Level of Foxp3 Expression

Differentiation, proliferation, and suppressive function or survival of Treg cells are influenced by various factors of energy metabolism. Therefore, the role of the Foxp3 protein in the regulation of cellular metabolism is also an important issue [127]. Naive T cells have modest metabolic requirements which are mainly related to the oxidation of pyruvate and fatty acids in the tricarboxylic acid (TCA) cycle [127]. However, when activated, the energy requirements of these cells increase. This is possible due to significant metabolic changes induced by the TCR receptor and costimulatory molecules such as phosphoinositol 3-kinase (PI3K) as well as AKT and rapamycin 1 (mTOR1) complexes, the activation of which are responsible for the regulation of genes responsible for the uptake and breakdown of glucose and other energetic compounds, including acids [127,128]. Such changes provide not only the energy needed for proliferation but also the necessary biosynthetic raw materials. According to conducted studies, inhibition of glycolysis may direct the differentiation of T CD4+ lymphocytes towards an anergic state which is then accompanied by an increased expression of the Foxp3 protein [128,129]. As a result of genetic or chemical ablation of mTOR and elimination of glycolysis facilitators, it causes the generation of iTreg cells compared to effector lines [130]. Additionally, the forced activation of AMP-activated protein kinase (AMPK), a regulator of lipid metabolism involved in T cell differentiation, leads to increased Foxp3 protein expression and iTreg cell differentiation [131,132]. Moreover, the use of fatty acid inhibitors such as etomixir or the carnitine palmitoyltransferase 1A inhibitor reduces the degree of differentiation of iTreg cells [133,134,135]. From studies conducted in recent years it can be concluded that the process of induction of Foxp3 protein expression by iTreg cells is extremely sensitive to metabolic factors [136]. In addition, mutations leading to inappropriate dominance of the T-lymphocyte glycolytic pathway destabilize the Treg cell phenotype and result in the loss of the ability to express the Foxp3 protein which then leads to the inability of cells to suppress inflammation [127,128,129].

### 3.5. Regulation of Foxp3 Protein Expression as a Potential Therapeutic Strategy for Autoimmune and Neoplastic Diseases

Treg lymphocytes, due to their extremely important functions in the human body related to the suppression of immune reactions, have now become an important research target for many scientists. Particularly noteworthy are those studies that concern the possibility of their introduction as a treatment for autoimmune diseases and cancers [137]. The results of research conducted mainly on animal models show that the use of compounds as inhibitors or modulators of Foxp3 protein expression may not only confirm the correctness of the hypotheses put forward by scientists but also discover these molecules with high clinical potential [138].

The first group of such compounds are inhibitors of HDAC enzymes which, as shown by studies, can enhance not only the expression of *FOXP3* but also participate in the process of increasing the number of Treg cells and their functions. Currently, these compounds have found their application in animal models as therapeutics used to regulate the activity of Treg cells in autoimmune diseases (e.g., colitis, prostatitis) [124,139] and organ transplantation processes [107] as well as in the treatment of certain neoplastic diseases (e.g., breast cancer, lymphoma) [140,141]. The studies available in the literature show that the use of HDAC inhibitors influences the induction of FOXP3 acetylation in Treg cells, which prompt changes in markers related to the activity and functioning of Treg cells themselves, including receptors for TNF-α, CTLA-4 or PD-1 and IL-10 [142]. Obviously, a full understanding of the mechanisms of action of HDAC inhibitors still requires a lot of intensive research before such treatment strategies can be introduced into widespread clinical use.

Another group of compounds with therapeutic potential are histone acetyltransferase inhibitors [100]. The available studies on animal models show that the use of small-molecule allosteric compounds, which may interact with the cofactors of the Foxp3 protein, contributes to the regulation of the function of this protein [100]. The analyses conducted so far focus on two compounds, Tip60 and p300, for which allosteric modifiers were developed [143]. In the case of Tip60, these compounds reduce the process of histone acetylation and induce association with the Foxp3 protein which has been used to treat autoimmune diseases (mainly colitis and collagen-induced arthritis) [144,145,146]. There are also studies on the use of Tip60 and p300 inhibitors for antitumor therapy—mainly prostate cancer [137,147]. Tip60 inhibitors have been shown to be able to inhibit the cancer cells’ growth by inducing the apoptosis process and to allow the reduction of Treg suppression without affecting the proliferation of T effector cells [148]. In the case of p300 inhibitors, their ability to suppress Treg function has been demonstrated [149].

In order to determine the best therapeutic strategy aimed at changing the level of Foxp3 protein expression many thorough studies should be carried out which will determine not only the mechanisms of action of selected allosteric molecules but also allow the evaluation of the effectiveness of such therapies in a clinical setting. However, therapies with the use of a targeted modification of the expression of the Foxp3 protein will certainly become a valuable tool in the future in the fight not only of autoimmune diseases but also of neoplastic diseases.

## 4. The Role of Foxp3 Protein in the Development of PIDs

**Figure 4:**
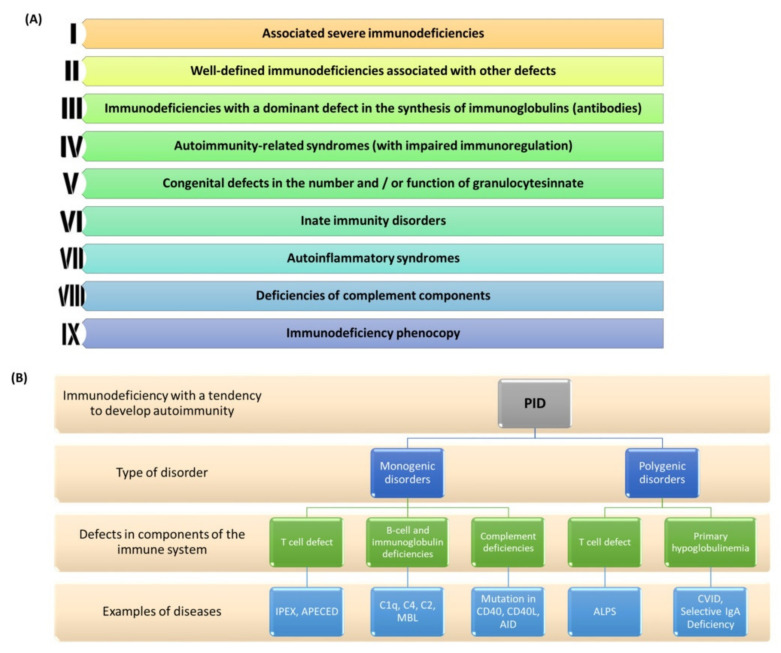
PID classification and division. (**A**) Updated PID classification by IUIS for 2019; (**B**) Division of PID based on the type of mono- and polygenic disorders with examples of diseases (prepared based on [156,157,158]). Abbreviations: APECED—Autoimmune polyendocrinopathy-candidiasis-ectodermal dystrophy; IPEX—immunodysregulation polyendocrinopathy enteropathy X-linked syndrome; C1q—complement component 1q; C4—Complement component 4; C2—Complement component 2; MBL—mannose-binding lectin; AID—Activation-induced cytidine deaminase; ALPS—Autoimmune Lymphoproliferative Syndrome; CVID—Common Variable Immunodeficiency; IgA—immunoglobulin A; CD40—cluster of differentiation 40; CD40L—cluster of differentiation 40 ligand.

PIDs are a group of genetically determined diseases that are characterized by the impairment of one or more mechanisms of innate or acquired immunity. Unlike secondary immunodeficiencies, the symptoms of PID occur throughout the patient’s life. This disease has a heterogeneous course, characterized by a wide spectrum of symptoms with varying severity which may manifest themselves in childhood or even in adulthood [150]. PIDs are genetic disorders that may be inherited (most often autosomal recessive) or appear for the first time in a given patient. According to the literature data presented by the International Union of Immunological Societies (IUIS), by 2019 over 406 diseases called PIDs and ~430 genes (the damage of which may lead to their development) have been described [151]. Individual mutations underlie the malfunction or lack of one or more elements of the immune system, including B and T lymphocytes, NK cells, phagocytes, or components of the complement system [151,152]. According to general estimates, the incidence of PID is in 1:2000 to 1:3000 live births [153,154]. Due to the possible occurrence of symptoms later in life, it is very difficult to determine the prevalence of PID in adults. Several reports conducted in various countries around the world show that diseases classified as PID occur in the population ranging from 1:8500 to 1:100,000 [155]. In the current literature we find that PIDs are classified into nine classes which were proposed and updated by the IUIS in 2019 (Figure 4A) [156]. Currently, there is a view in the literature that some PIDs may have a multi-gene basis because only the presence of defects of several genes at the same time is clinically manifested. In addition, the presence of a PID phenocopy may also be the result of an autoimmune reaction against certain components of the immune system itself (e.g., against certain interferons) (Figure 4B) [157].

The course of PIDs varies considerably which means that many clinical symptoms may be present from birth or may gradually worsen over time until the disease develops in childhood or adulthood. According to the literature, the mean time from the onset of symptoms to full PID diagnosis is on average ~5 years and largely depends not only on the type of deficiency and the patient’s age but also on the patient’s country of origin [159,160]. This is due to the lack of awareness and education in society as well as the lack of preparation in doctors to make accurate and quick diagnoses. Therefore, it is extremely important to search for diagnostic markers that allow for the shortening of time for accurate diagnosis as well as a comprehensive analysis of genetic disorders contributing to the development and progression of many diseases classified as PID [161,162] One such molecule is the Foxp3 protein in which mutation causes the immunodysregulation polyendocrinopathy enteropathy X-linked syndrome (IPEX) classified as a monogenic disorder [163]. Additionally, recent research sheds new light on the role of this protein in the development of CVID (common variable immunodeficiency) which is an example of a polygenic disorder. The *FOXP3* transcription factor is expressed on CD4+ Treg cells and is crucial for Treg function which is responsible for suppressing immune responses, especially at their early stages. The most important function of Foxp3 is its ability to confer suppressive activity on Treg cells, for example, by maintaining constitutive high expression of CTLA-4. It acts by preventing the activation and proliferation of B and T lymphocytes [164]. However, Foxp3 alone does not control all aspects of Treg biology and is not the initiating factor in Treg development [165,166]. The importance of Treg cells has been demonstrated in murine models—depletion of Foxp3+CD4+ Treg cells resulted in severe autoimmunity, allergy, and immunopathology (e.g., IPEX) in otherwise normal animals and those same diseases can be prevented by reconstituting Treg cells [167,168,169]. Moreover, it has also been demonstrated that Treg cells could prevent the progression of and even cure established autoimmune/inflammatory diseases [170,171]. They also play an important role in allergy prevention [172]. Foxp3+ Treg cells likewise depend on a lot of other suppressive molecules, such as IL-10, TGF-β, CD39, CD73, IL-35, and TIGIT, for their inhibitory function; most of which work by suppressing autoimmunity [173].

### 4.1. The Role of Mutations within the Foxp3 Protein in PID Development

The studies conducted so far indicate the presence of 63 identified mutations within the *FOXP3* gene which affect its proper functioning and contribute to the development of autoimmune diseases [174,175]. The vast majority of the discovered mutations concern the fork-head binding domain of the Foxp3 protein that influences the processes of nuclear import and DNA binding which are necessary for the suppressive activity of this protein. Some of the mutations were also found in the leucine zipper region which impaired the dimerization function of the Foxp3 protein; other mutations influenced the spatial change in the structure and position of the domains within the protein or led to a decrease in mRNA stability for the *FOXP3* gene [174,175,176].

#### 4.1.1. The Role of Foxp3 Protein in IPEX Development

**Figure 5:**
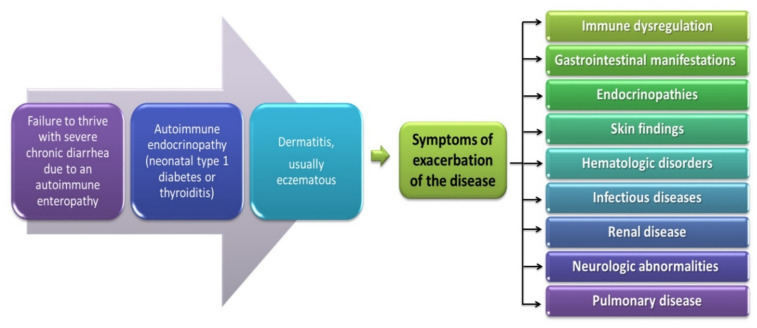
Symptoms of IPEX developed from [177].

The occurrence of mutations in the *FOXP3* gene is associated with the development of the IPEX disease syndrome and was described for the first time in 1982 by Powell et al. as a rare immunodeficiency syndrome with a genetic predisposition [176]. This disease is characterized by the presence of three specific clinical symptoms such as enteropathy with chronic diarrhea (most often acute, watery, and bloody diarrhea), endocrinopathy (insulin-dependent diabetes type I), and dermatitis (Figure 5) [35]. IPEX is a recessive disorder related to the X chromosome; therefore, it occurs only in males (in the first six months of life) and causes T lymphocyte activation, accompanied by the overproduction of cytokines, and leads to autoimmune disorders with the presence of various autoantibodies [177]. This results in the development of many serious diseases such as type 1 diabetes and autoimmune hemolytic anemia as well as hypopituitarism or thyroid gland disorders and numerous skin lesions such as eczema. In many patients, exacerbation of the disease is also observed and caused by infections or food allergens. Subsequently, the main symptoms of the gastrointestinal tract or skin lesions are intensified or exacerbated as well as other disorders related to kidney, digestive, and immune system diseases (Figure 5) [163,178]. Due to such a wide range of symptoms, patients without appropriate treatment (immunosuppressants and bone marrow transplantation) die before reaching the age of 2 [176].

The assessment of the presence of the IPEX syndrome requires the use of many extensive, basic, and specialized tests aimed at a correct diagnosis. Basic tests include a complete blood count with smear, determination of serum glucose concentration, thyroid function, and immunoglobulin levels as well as food hypersensitivity tests and the percentage of individual cells of the immune system, especially T and B lymphocytes (Table 5). Based on the obtained results of basic tests, advanced tests are ordered, including endoscopy with intestinal biopsy, skin biopsy, immunophenotyping of Treg lymphocytes, and sequencing of the *FOXP3* gene (Table 5) [177].

Genetic conditions are related to the mutation within the transcription factor Foxp3 which was found in nearly 60% of patients with IPEX syndrome. These include missense or frame alteration mutations and insertion or deletion mutations at splicing sites which result in the loss of function of this protein. The occurring mutations also have consequences regarding the quantitative and functional disorders of Treg cells in the body deficiencies which cause autoimmune disease. We can find a description in the literature of most cases where mutations occurring within the Foxp3 protein are hereditary, although this does not exclude the occurrence of mutations in a sporadic manner [185,186,187]. These mutations take place in almost every region encoding the Foxp3 protein which clearly indicates that each domain of this protein is functional and necessary for its proper functioning. Although mutations in the non-coding regions are also known, their frequency is much lower compared to the mutations in the coding regions, however, their occurrence may also be the cause of the development of IPEX [28,188]. Nevertheless, scientists have not shown a clear correlation between genotype and phenotype [181,189,190].

An important diagnostic and cognitive tool turned out to be the use of flow cytometry which makes it possible to assess the expression of Foxp3 on cells of the immune system. Although high levels of *FOXP3* expression are commonly attributed to CD4+CD25_high_ Treg cells, it is also induced by TCR stimulation in naive CD4+CD25− T cells and may persist for up to several days in activated CD4+ T cells [191]. The belief of many scientists that subsets of CD4+ T cells expressed in different tissues have different patterns of CD25 and other Treg cell markers further complicates the task of distinguishing Treg *FOXP3*+ from *FOXP3*+ found on other cells of the immune system. Therefore, it is necessary to search for new diagnostic markers to allow their safe differentiation. One such marker is the IL-7 receptor (CD127) promoter which is the target of *FOXP3* mediated transcriptional repression. Thanks to this discovery, scientists were able to identify the most suppressive population of human Treg cells, expressing the phenotype CD4+CD25+CD127_low_ [192,193]. This has been used in the diagnostic methodology of the IPEX team where cells identified as CD4+CD25+CD127_low_ correlate well with the population of CD4+CD25+*FOXP3*+ cells. Of course, the two populations should not be considered identical since conventional CD4+CD25-CD127+ T (Tcon) cells rapidly decrease CD127 expression following IL-7 signaling and TCR stimulation [194]. Studies have shown that cells with the CD4+CD25+CD127_low_ phenotype may also be present in some IPEX patients who suffer from decreased FOXP3 expression due to the occurrence of hypomorphic mutations [55,195]. It has also been demonstrated that patients with IPEX syndrome who have missense mutations and deletions in splicing sites do not have Treg CD4+CD25+Foxp3+ lymphocytes and have a more severe form of the disease. Additionally, the absence of CD4+CD25+ cells confirms the diagnosis [35]. IPEX also has high levels of IgE and IgA immunoglobulins, as well as eosinophilia, which proves that the transcription factor Foxp3 is strongly associated with the human immune response [179,180,181,182].

Due to the special role of the Foxp3 protein in the development and progression of IPEX teams, many scientists are considering using this molecule as a therapeutic agent in the treatment of this disease. The studies available in the literature also show that in the absence of antigenic stimulation, *FOXP3* expression is promoted in CD4+CD25− T cells by signaling cytokines, which include STAT5, IL-2, IL-7, and IL-15, on peripheral blood cells [35,196,197]. Additionally, it has been shown that cytokine treatment does not induce *FOXP3* expression in lymph node derived CD4+ T cells [35,197]. The use of flow cytometry analysis has shown that the expression level of the Foxp3 protein on TCD4+ lymphocytes from peripheral blood can vary up to 40-fold between individual cells. This explains that the strength and duration of *FOXP3* induction may influence discrepancies in cytokine activation-induced T cell suppression [35,198,199,200,201].

**Figure 6:**
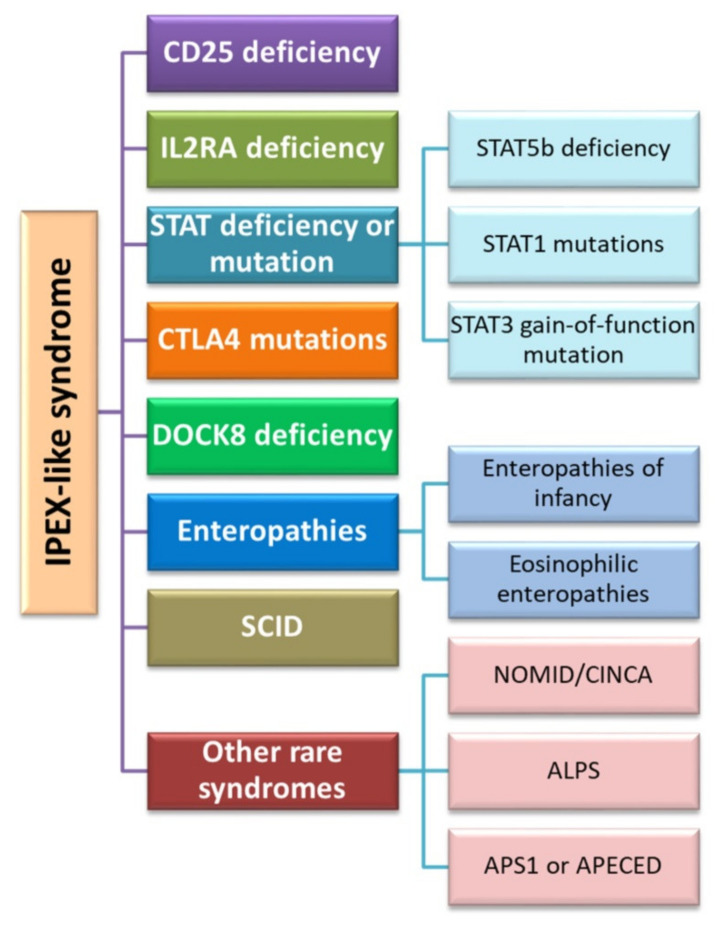
Diseases classified into IPEX-like syndromes (based on [177]). Abbreviations: IL2RA—Interleukin 2 Receptor Subunit Alpha; STAT—signal transducer and activator of transcription protein; STAT5—Signal Transducer And Activator Of Transcription 5; STAT1—Signal transducer and activator of transcription 1; STAT3—Signal Transducer And Activator Of Transcription 3; CTLA4—cytotoxic T cell antigen 4; DOCK8—Dedicator of cytokinesis 8; SCID—severe combined immunodeficiency; NOMID—neonatal onset multisystemic disease; CINCA—chronic infantile neurological, cutaneous, and articular syndrome; ALPS—Autoimmune Lymphoproliferative Syndrome; APS-1—Autoimmune Polyglandular Syndrome Type 1; APECED—Autoimmune polyendocrinopathy-candidiasis-ectodermal dystrophy.

Another therapy using the role of the Foxp3 protein proposed in the literature is the use of hematopoietic stem cell transplantation. Currently, this therapy is the only treatment option for sick patients with IPEX. According to the study by Passerini et al. in 2013, the conversion of CD4+ Treg cells after lentivirus-mediated transfer of the *FOXP3* gene results in a population of CD4+*FOXP3*+ T cells which exhibit a stable phenotype and preserved suppressive function. In addition, their studies have shown that CD4+*FOXP3*+ T cells are stable during inflammation not only in vitro but also in in vivo models. Therefore, based on the above observations, the researchers proposed the use of *FOXP3* gene transfer therapy in the IPEX syndrome in order to restore immune tolerance [202]. From the description of clinical cases and literature data, we can conclude that there are also patients with symptoms that resemble IPEX syndrome, including enteropathy, autoimmune endocrinopathy, and dermatitis, but the age and gender of these patients are more varied (these symptoms are also present in women) [203]. From the study by Ochs and Torgerson on 100 patients with a phenotype corresponding to IPEX syndrome, nearly half of them did not have a mutation in the *FOXP3* transcription factor gene [186]. Such diseases include CD25 or IL-2RA deficiency, mutations within STAT5b, STAT1 or STAT3, Dedicator of Cytokinesis 8 (DOCK8) deficiency as well as infantile or eosinophilic enteropathies and severe combined immunodeficiency (SCID) (Figure 6). The underlying genetic defect of these syndromes is unknown and requires further intensive research.

#### 4.1.2. The Role of the Foxp3 Protein in the Development of CVID

Recent studies have also shown the role of the Foxp3 protein in the development of another PID disease, i.e., CVID. This disorder belongs to the humoral immunity deficiencies and is characterized by a relatively mild course. It is a type of polygenic disorder associated with the defective production of immunoglobulins, often accompanied by autoimmunity [204]. The epidemiology of this disorder is still difficult to define. Literature data show that the estimated prevalence of CVID in the population is 1 in 30,000 people. The diagnosis of CVID occurs in most patients between the age of 20 and 40 due to late symptoms [205,206]. Based on the analysis carried out by Grathman et al., in 2014, it was possible to establish the relationship between the clinical picture and the differences and effects of immunoglobulin treatment in 2212 patients from several European countries. The results of this review showed that patient survival depends on the time of diagnosis as well as the age when the first symptoms of the disease appeared [207]. The later the first symptoms appeared and the more delayed diagnosis of the disease, the greater the risk of death at any age (each year of delay in diagnosis increases the risk of death by about 4.5%) [207]. However, the etiology of CVID is not fully known, as only 20% of patients have the genetic cause identified. The most common form is sporadic cases with no family history of the disease (90%) [208]. They can be caused by a complex interaction of environmental and genetic components (multi-factor inheritance), but genes involved in the development and function of immune cells have now been shown to be the main cause [208,209]. As we know, the main role of the immune system is to defend against infections while protecting the body’s own cells. Antibodies, also known as immunoglobulins, are proteins produced by B lymphocytes. In order for B lymphocytes to function effectively, they usually need the help of other immune cells such as T lymphocytes. Most people with CVID have a normal number of B cells, but this is characterized by a maturation disruption and thus a decrease in antibody synthesis. These disorders can be caused by a lack of needed help from T cells to develop a normal immune response [210,211,212]. As a result, people suffering from CVID will differ in their ability to elicit effective antibody responses due to the lowered levels of immunoglobulin. We can distinguish three types of disorders here; the first one concerns the three main types of immunoglobulins (IgG, IgA, and IgM), the second one concerns disorders within IgG and IgA, and the third group concerns those in which only IgG is lowered [213,214]. A diagnosis of CVID is usually confirmed by abnormal blood tests and medical history.

Due to the important role of T lymphocytes in stimulating the synthesis of antibodies by B lymphocytes, some scientists have started research to determine whether the Foxp3 protein may be involved in this process [215]. The research conducted by Horn et al. in 2009 shows that this protein may indirectly influence the development of CVID. They analyzed the percentage of CD4+ Treg lymphocytes among patients diagnosed with CVID from different cities/countries: Freiburg, London, and Sydney, and correlated it with clinical symptoms. The percentage of Treg cells defined as CD25+Foxp3+ and CD25+CD127_low_Foxp3+ or CD25+CD127_low_CD4+ was analyzed and the results were compared with data from healthy patients. They found that, regardless of the phenotype used to define them, patients with CVID experienced a significant decrease in the percentage of Treg cells which correlated with the development of autoimmune disease. This provided evidence that a reduction in the number of Treg cells in CVIDs may play a role in the development and progression of clinical symptoms and may also contribute to understanding the pathogenesis of CVID complications [216]. Other research by Genere et al. built on the work presented by previous researchers; they showed that patients diagnosed with CVID and autoimmune disease had a significantly reduced frequency of CD4+CD25_HIGH_Foxp3+ cells in the peripheral blood, accompanied by a reduced intensity of FOXP3 expression. Additionally, they found that although CVID patients with autoimmunity had a reduced frequency of CD4+CD25_HIGH_Foxp3+ cells, FOXP3 expression levels did not differ from those of healthy controls. Thanks to the obtained results, the researchers showed that CD4+CD25_HIGH_Foxp3+ cell homeostasis is disturbed in patients with CVID, especially in the presence of autoimmunity, which may indicate that Treg lymphocytes are involved in the pathological mechanisms of CVID [217]. The results of these studies were also confirmed by another research team (Arandi et al., 2013) which showed that the frequency of Treg was significantly lower in patients with CVID than in healthy subjects and that in patients with CVID, in whom autoimmunity was detected, the percentage of cells analyzed is significantly reduced compared to the cases without autoimmune diseases. There was also a significant difference in the expression level of the Foxp3 factor between patients with CVID and the control group [218]. The reduction of the expression level of the Foxp3 protein in patients with CVID was also analyzed and confirmed by Yu et al. [52]. They showed that the reduction in the levels of FoxP3, granzyme A, and pStat5 was significantly correlated with the degree of Treg dysfunction in CVID [52]. However, a full understanding of the role of Foxp3 in the development of CVID requires further extensive interdisciplinary research to understand its role in the pathogenesis of primary immunodeficiencies.

## 5. Materials and Methods

### 5.1. Search Strategy, Study Selection, and Data Extraction

The literature analysis was carried out on the PubMed database where the search for available articles was performed based on the following keywords: “Foxp3”, “IPEX”, “IPEX like”, “Immunodysregulation, Polyendocrinopathy, and Enteropathy, X-Linked”, “CVID”, “Common Variable Immunodeficiency”, and “Primary Immunodeficiency”. The time range of the searched articles was established for the years 2000 to 2021 and filters related to the type of articles (clinical trials, review, systematic review) were used. Repetitions were rejected from the found articles. The suitability for the inclusion of each work into the publication was thoroughly assessed. Eventually, 223 articles were included in the review.

### 5.2. Biostatistical Analysis

For bioinformatic analysis, the amino acid sequences deposited in the UniProt database [219] were used. The identification numbers of the Foxp3 protein and its three isoforms, along with their amino acid sequences, are provided in Supplementary Materials Table S1. These sequences were used to carry out further bioinformatic analyses. The sequence length and molecular weight of individual proteins were pulled from the UniProt database. The determination of the isoelectric point of tested interleukins and their amino acid composition was carried out using the IPC isoelectric point calculator software available online [220]. The analysis of the second-order structure of interleukins was carried out using the NetSurfP-2.0 online program [221]. The amino acid sequences from the UniProt databases (Supplementary Materials Table S1) were used to analyze the identity of the amino acid sequences of the Foxp3 protein and its isoforms. The amino acid sequences of individual proteins were compared with each other using the Clustal Omega program available on their website [222]. The results of the analyses were presented as the percentage of identical amino acids in the analyzed amino acid sequences.

## 6. Conclusions

Numerous studies conducted in recent years have shown that Treg lymphocytes, which express Foxp3, appear in the human body immediately after birth and lead to the development of many inflammatory and autoimmune diseases after they are depleted. The Foxp3 protein has been shown to be necessary for lymphocytes in the thymus to differentiate into Treg lymphocytes. High expression of this transcription factor also guarantees their suppressive effect. The Foxp3 protein influences several cellular processes both directly and indirectly. In the process of cytokine production regulation, the Foxp3 protein interacts with numerous proteins and transcription factors such as NFAT, nuclear factor kappa B, and Runx1/AML1, and is involved in the process of histone acetylation in condensed chromatin. Thanks to their analyses and many experiments, scientists have shown that the similarity in the disturbance of the functioning of the FOXP3 gene in humans and mice is very similar. This allows for the conclusion that the process of dominant self-tolerance in these organisms is similar to each other. Scientists’ persistence in researching the Foxp3 protein has led to including this factor in one of the most reliable molecular markers of natural Treg lymphocytes. In addition, studies on the dysfunction of the Foxp3 transcription factor caused by the mutagenesis process have shown that it significantly affects disorders of the immune response as well as the development and progression of primary immunodeficiencies or autoimmune diseases.

## Figures and Tables

**Table 1:** Characteristics of the basic properties of the Foxp3 protein and its isoforms.

Name	Amino Acid Length	Mass [kDa]	Isoelectric Point	Amino Acid Composition		Secondary Structure		Protein ID
				Hydrophobic Amino Acids [%]	Hydrophilic Amino Acids [%]	α-Helix	β-Starnad	
Foxp3	431	47.24	8.62	53.60	46.40	12 (26.15%)	10 (6.26%)	Q9BZS1
Isoform2	396	43.41	8.53	52.27	47.73	10 (25.25%)	9 (5.55%)	Q9BZS1-2
Isoform 3	456	49.84	8.00	53.51	46.49	14 (27.41%)	10 (6.56%)	Q9BZS1-3
Isoform 4	404	44.41	8.52	53.47	46.53	12 (24.50%)	11 (7.43%)	Q9BZS1-4

**Table 2:** The degree of identity of the amino acid sequence of the Foxp3 protein and its isoforms.

	Foxp3	Isoform 2	Isoform 3	Isoform 4
Foxp3	-	91.88%	80.65%	93.73%
Isoform 2	91.88%	-	86.84%	85.61%
Isoform 3	80.65%	86.84%	-	75.15%
Isoform 4	93.73%	85.61%	75.15%	-

**Table 3:** Treg lymphocyte subpopulations (based on [70]).

Name of the Subpopulation		Characteristics
Expressing Foxp3	T lymphocytes CD4+CD25+Foxp3+	The most widely studied and characterized sub-population of regulatory cells. Characterized by the expression of the Foxp3 transcription factor and accompanied by a high expression of the CD25 surface molecule.
	T lymphocytes CD8+CD25+Foxp3+	A subpopulation of CD8+ T cells that are much less well understood than CD4+Foxp3+ cells.
Not expressing Foxp3	Type 1 regulatory T cells (Tr1)	Cells with the CD4+Foxp3− phenotype secreting significant amounts of IL-10.
	Th3 lymphocytes	CD4+Foxp3− cells secreting significant amounts of TGF-β.
	CD8+CD28− lymphocytes	identified with pre-Ts lymphocytes that also do not express Foxp3.

**Table 5:** Meanings of basic and specialized tests in the diagnosis of IPEX.

Type of Research	Type of Examination	The Importance of the Examination	Reference
Basic examination	Complete blood count with a smear	Presence of eosinophilia, neutropenia, anemia, or thrombocytopenia	[177]
	Serum glucose concentration	Glucose monitoring can help to detect the presence of type 1 diabetes	[177]
	Functioning of the thyroid gland	Elevated levels of anti-thyroid antibodies	[177]
	Concentration of immunoglobulins	Increase in IgE level in most patients, increase in IgA level in half of the patients, normal IgG and IgM	[178,179,180,181,182]
	Food hypersensitivity test	Presence of IgE-dependent food allergy	[181,183]
	Determination of the percentage of T and B lymphocytes	T and B cell subsets are usually normal	[177]
Specialized research	Endoscopy with intestinal biopsy	Necessary to characterize the presence of enteropathy of the small intestine, combined with the performance of Foxp3 staining	[184]
	Skin biopsy	The presence of lymphocyte infiltrates in biopsy samples as an autoimmune process	[177]
	Treg lymphocyte immunophenotyping	Determines the amount of Treg and the expression level of Foxp3	[177]
	Sequencing of the *FOXP3* gene	Evaluates mutations within the *FOXP3* gene to confirm the clinical picture	[177]

## Data Availability

Not applicable.

## Supplementary Materials

The following are available online at https://www.mdpi.com/article/10.3390/jcm11040947/s1, Supplementary Materials Table S1: Amino acid sequences of the Foxp3 proprotein and its three isoforms from the UniProt database.
