# Supplementary material for: The Importance of the Transcription Factor Foxp3 in the Development of Primary Immunodeficiencies
Source: J Clin Med. 2022 Feb 11;11(4):947. doi: 10.3390/jcm11040947 (PMC8874698; doi:10.3390/jcm11040947)
Supplement: Supplementary file 1 [file jcm-11-00947-s001.zip › jcm-1559584-supplementary.pdf]

Supplemantary Materials S1- Amino acid sequences of the Foxp3 protein and its three isoforms from the UniProt database

Name	Protein ID	Sequence
**Foxp3**	**Q9BZS1**	MPNPRPGKPSAPSLALGPSPGASPSWRAAPKASDLLGARGPGGTFQGRDLRGGA<br>HASSSSLNPMPPSQLQLPTLPLVMVAPSGARLGPLPHLQALLQDRPHFMHQLSTV<br>DAHARTPVLQVHPLESPAMISLTPPTTATGVFSLKARPGLPPGINVASLEWVSREPA<br>LLCTFPNPSAPRKDSTLSAVPQSSYPLLANGVCKWPGCEKVFEEPEDFLKHCQADH<br>LLDEKGRAQCLLQREMVQSLEQQLVLEKEKLSAMQAHLAGKMALTKASSVASSD<br>KGSCCIVAAGSQGPVVPAWSGPREAPDSLFAVRRHLWGSHGNSTFPEFLHNMDY<br>FKFHNMRPPFTYATLIRWAILEAPEKQRTLNEIYHWFTRMFAFFRNHPATWKNAI<br>RHNLSLHKCFVRVESEKGAVWTVDELEFRKKRSQRPSRCSNPTPGP
**Isoform 2**	**Q9BZS1-2**	MPNPRPGKPSAPSLALGPSPGASPSWRAAPKASDLLGARGPGGTFQGRDLRGGA<br>HASSSSLNPMPPSQLQLSTVDAHARTPVLQVHPLESPAMISLTPPTTATGVFSLKAR<br>PGLPPGINVASLEWVSREPALLCTFPNPSAPRKDSTLSAVPQSSYPLLANGVCKWP<br>GCEKVFEEPEDFLKHCQADHLLDEKGRAQCLLQREMVQSLEQQLVLEKEKLSAM<br>QAHLAGKMALTKASSVASSDKGSCCIVAAGSQGPVVPAWSGPREAPDSLFAVRR<br>HLWGSHGNSTFPEFLHNMDYFKFHNMRPPFTYATLIRWAILEAPEKQRTLNEIYH<br>WFTRMFAFFRNHPATWKNAIRHNLSLHKCFVRVESEKGAVWTVDELEFRKKRSQ<br>RPSRCSNPTPGP
**Isoform 3**	**Q9BZS1-3**	MPNPRPGKPSAPSLALGPSPGASPSWRAAPKASDLLGARGPGGTFQGRDLRGGA<br>HASSSSLNPMPPSQLQLSTVDAHARTPVLQVHPLESPAMISLTPPTTATGVFSLKAR<br>PGLPPGINVASLEWVSREPALLCTFPNPSAPRKDSTLSAVPQSSYPLLANGVCKWP<br>GCEKVFEEPEDFLKHCQADHLLDEKGRAQCLLQREMVQSLEQQLVLEKEKLSAM<br>QAHLAGKMALTKASSVASSDKGSCCIVAAGSQGPVVPAWSGPREAPDSLFAVRR<br>HLWGSHGNSTFPEFLHNMDYFKFHNMRPPFTYATLIRWAILEAPEKQRTLNEIYH<br>WFTRMFAFFRNHPATWKVSSSEVAVTGMASSAIAAQSGQAWVWAHRHIGEERD<br>VGCWWWLLASEVDAHLLPVPGLPQNAIRHNLSLHKCFVRVESEKGAVWTVDEL<br>EFRKKRSQRPSRCSNPTPGP
**Isoform 4**	**Q9BZS1-4**	MPNPRPGKPSAPSLALGPSPGASPSWRAAPKASDLLGARGPGGTFQGRDLRGGA<br>HASSSSLNPMPPSQLQLPTLPLVMVAPSGARLGPLPHLQALLQDRPHFMHQLSTV<br>DAHARTPVLQVHPLESPAMISLTPPTTATGVFSLKARPGLPPGINVASLEWVSREPA<br>LLCTFPNPSAPKDSTLSAVPQSSYPLLANGVCKWPGCEKVFEEPEDFLKHCQADHL<br>LDEKGRAQCLLQREMVQSLEQQASSDKGSCCIVAAGSQGPVVPAWSGPREAPDSL<br>FAVRRHLWGSHGNSTFPEFLHNMDYFKFHNMRPPFTYATLIRWAILEAPEKQRTL<br>NEIYHWFTRMFAFFRNHPATWKNAIRHNLSLHKCFVRVESEKGAVWTVDELEFR<br>KKRSQRPSRCSNPTPGP
